# Supplementary material for: Enhanced detection of RNA modifications in Escherichia coli utilizing direct RNA sequencing
Source: Cell Rep Methods. 2025 Sep 9;5(9):101168. doi: 10.1016/j.crmeth.2025.101168 (PMC12539251; doi:10.1016/j.crmeth.2025.101168)
Supplement: Document S1. Figures S1–S7 [file mmc1.pdf]

Cell Reports Methods, Volume *5*

Supplemental information

Enhanced detection of RNA modifications in *Escherichia coli* utilizing direct RNA sequencing

Zhihao Guo, Yanwen Shao, Lu Tan, Beifang Lu, Xin Deng, Sheng Chen, and Runsheng Li

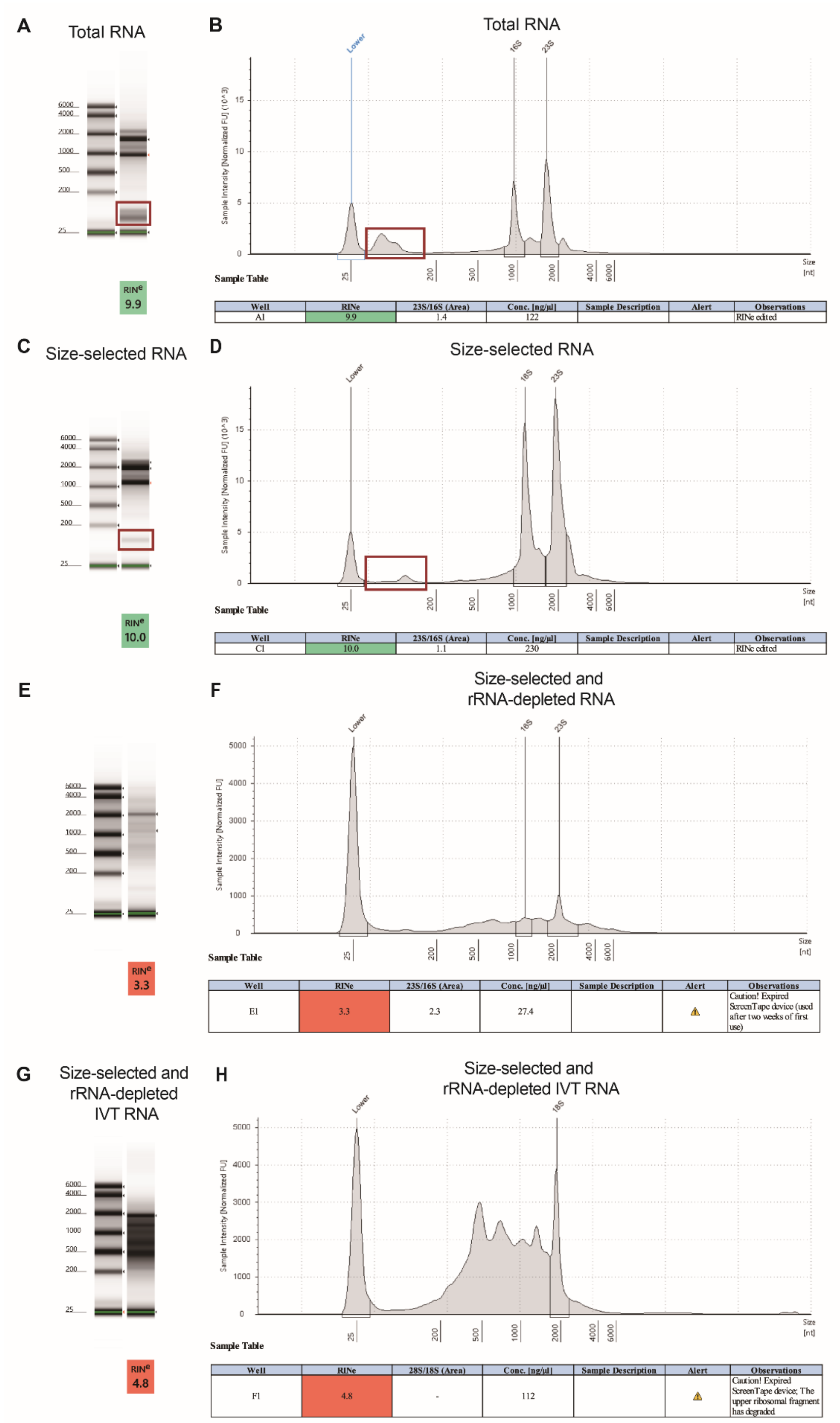

Figure S1. The TapeStation gel plots of the K12 RNA samples. Related to Result "The RNA004 kit improved sequencing yield and quality for bacterial RNA". (**A** and **B**) are total RNA, (**C** and **D**) are size selection RNA, (**E** and **F**) are size selection and ribosome depletion (ss&rd) RNA, and (G and H) are IVT RNA. IVT RNA was generated based on ss&rd_RNA.

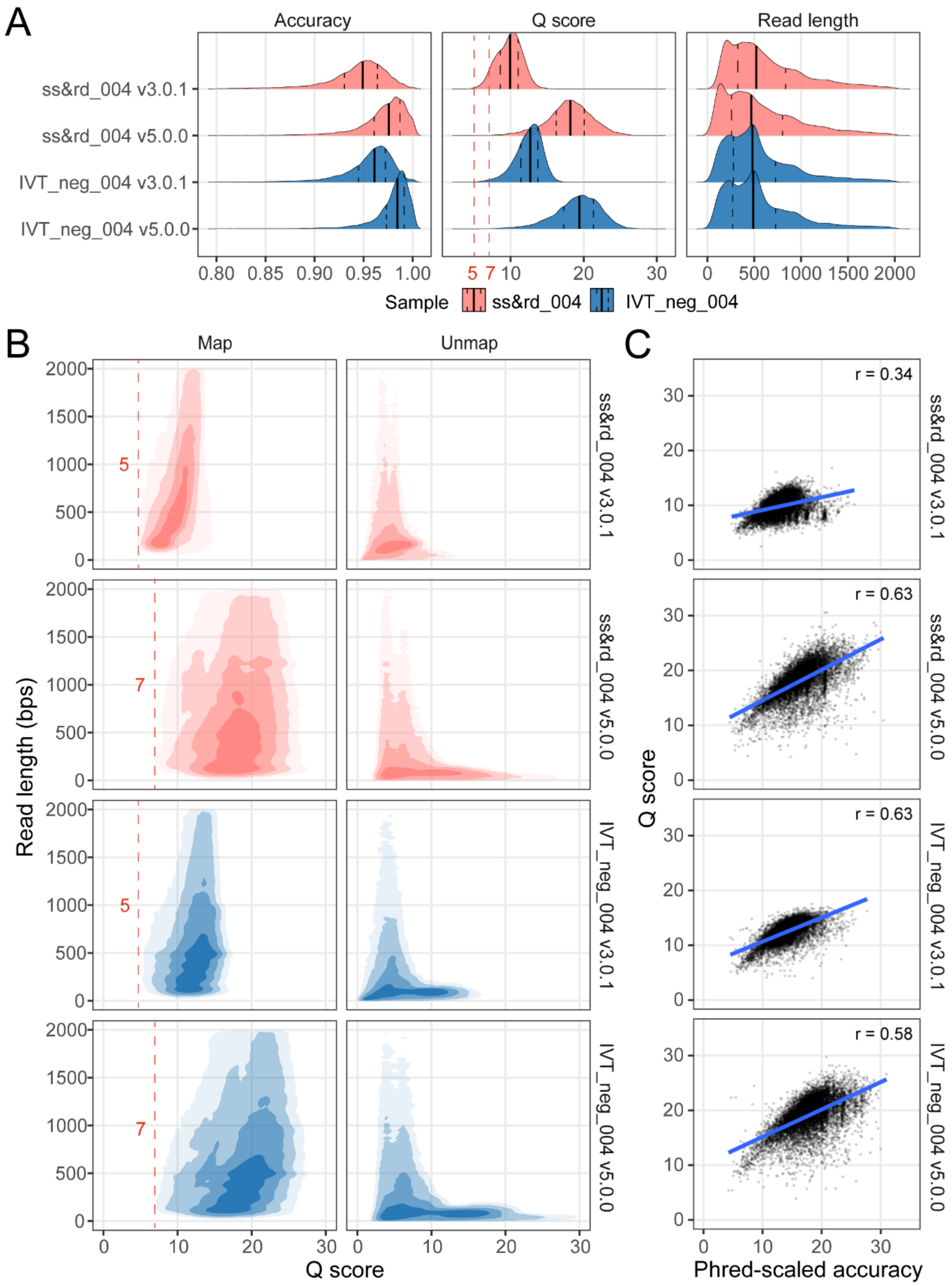

Figure S2. Raw read features comparison between different Dorado basecalling model. Related to Figure 1. (**A**) Mapping accuracy, Q scores, and read lengths of mapped reads across different basecall model versions. A Q score of 5 is an effective filter for version 3.0.1, while a Q score of 7 is optimal for version 5.0.0. **(B)** Relationship between read length and the quality of mapped and unmapped reads, presented as a 2D density plot. Read quality is indicated by Q scores. **(C)** Correlation between Q scores and Phred-scaled mapping accuracy across different samples and model versions.

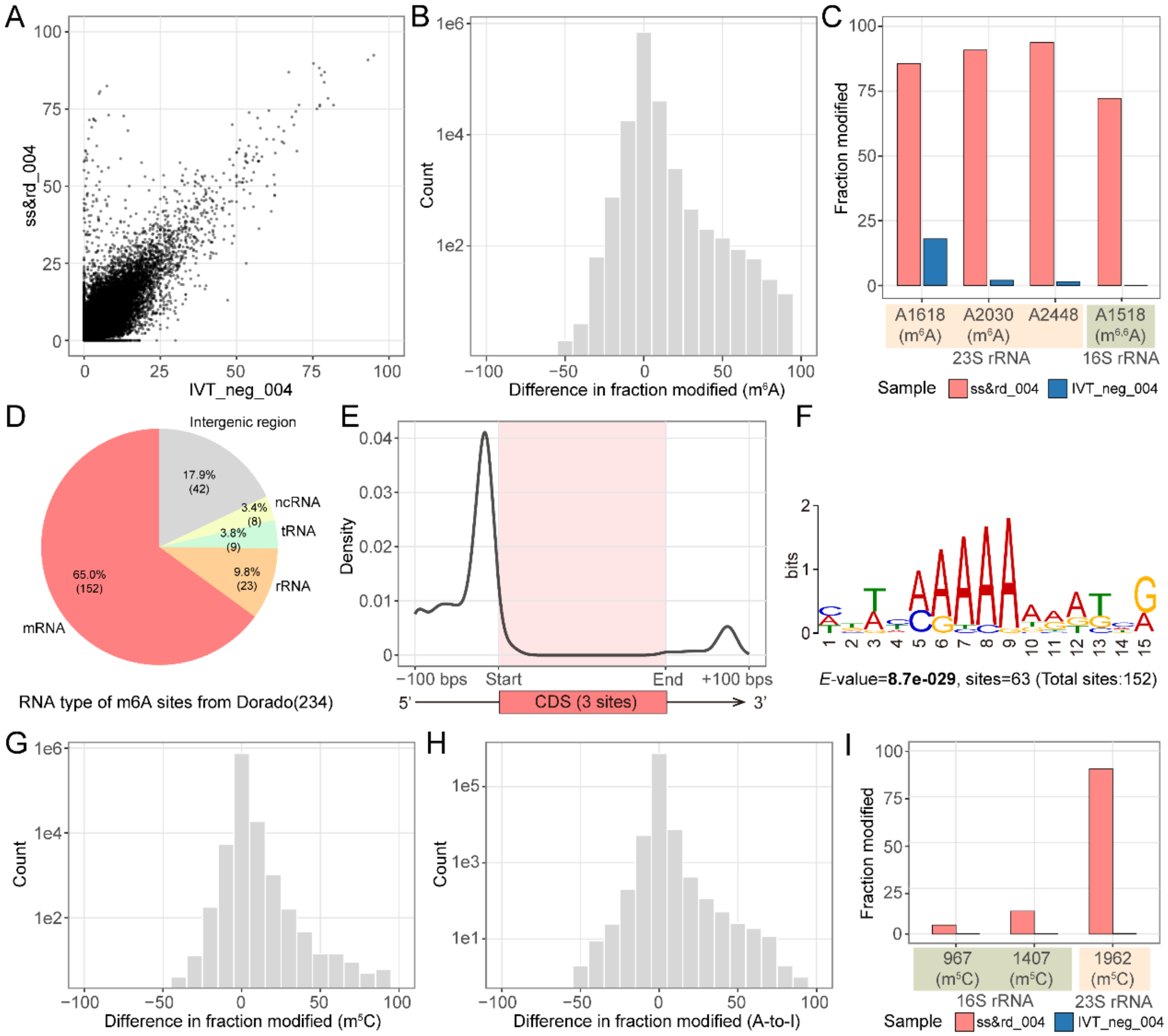

Figure S3. Evaluation and characterization of Dorado modification detection model. Related to Figure 2. **(A)** The dot plot illustrates the "fraction modified" values for each position detected by the m6A all-context model across both samples. **(B)** The right-skewed distribution of the difference in "fraction modified" (WT - IVT) is presented, with the y-axis displayed in log10 scale. **(C)** The "fraction modified" values exceeding 65 for E. coli rRNA are highlighted, including two known m6A sites on 23S rRNA (A1618, A2030). After applying a filter of 65, **(D)** Pie plot shows the proportion and number of high-confidence sites within the expanded annotation region. **(E)** The density distribution around the CDS region reveals three sites located within the CDS. **(F)** Motif enrichment analysis using MEME indicates a notable enrichment primarily at the 5' end of the UTR polyA region. **(G)** and **(H)** present the distribution of the difference in "fraction modified" (WT - IVT) for the m5C model and the A-to-I model, respectively. Finally, **(I)** Bar plots illustrate the performance of the m5C model on three known m5C sites within rRNA.

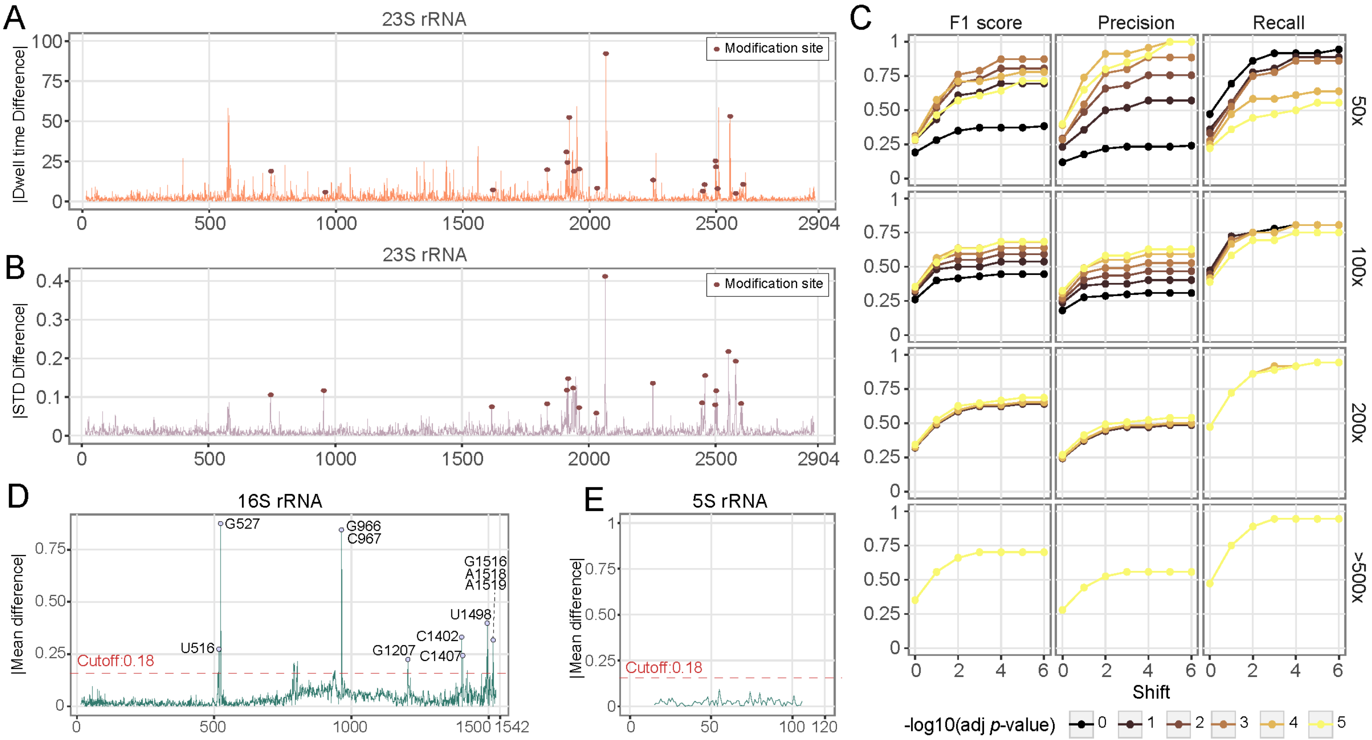

Figure S4. Exploration of statistical features of the nanoSundial. Related to Figure 3. **(A)** and **(B)** display the absolute differences in dwell time and standard deviation on 23S rRNA. **(C)** Dot and line plots present the F1 score, precision, and recall values after applying filters based on mean and dwell time. Effects of adjusted *p*-values and shifts were displayed across different coverage levels. Once the coverage reaches a certain threshold (>200x), the *p*-value cutoff becomes ineffective. **(D)** and **(E)** represent the absolute differences in mean values for 16S and 5S rRNA.

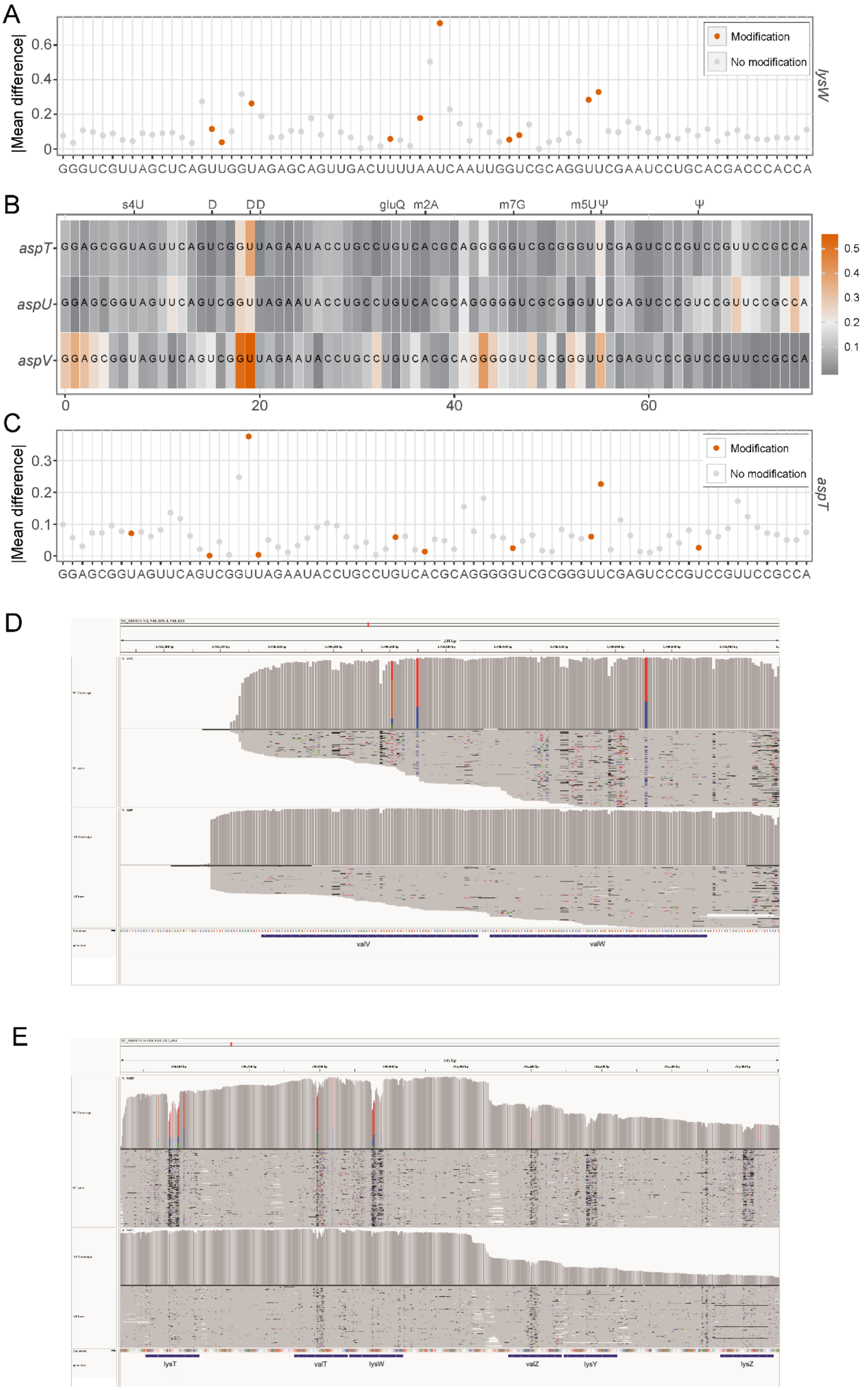

Figure S5. Profiling tRNA modification patterns. Related to Figure 4. **(A)** showcased the absolute value of mean difference on gene *lysW*. **(B)** Heat maps illustrate the absolute value of mean difference

for the paralogs of tRNA aspartate (asp). The colors at each position represent the magnitude of these absolute differences. **(C)** A showcase of the absolute value of mean difference on gene *aspT*. **(D)** and **(E)** are IGV Screenshots of tRNA Regions. **(D)** is showcase of *valV* and *valW*, and **(E)** is showcase of *lysT*, *valT*, *lysW*, *valZ*, *lysY* and *lysZ*

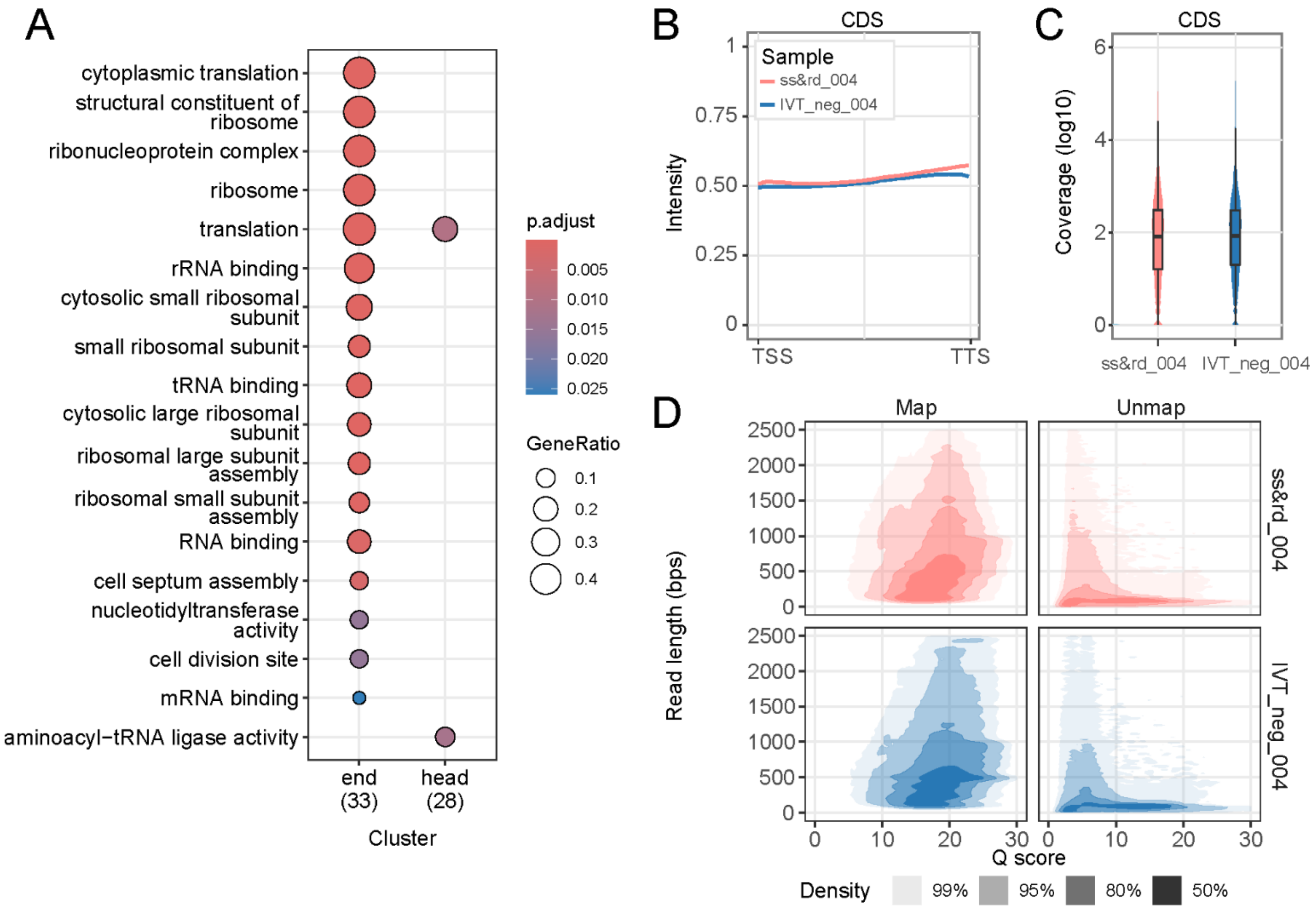

Figure S6. Genomic and sequencing analysis between Wild-Type (WT) and In Vitro Transcribed (IVT) Samples. Related to Figure 6 and Discussion. **(A)** GO pathways enrichment of the stably modified genes according to the position in the operons, namely the start or end of the operon. **(B)** Average coverage intensity across the CDS from start to end. The y-axis represents Intensity, calculated as the depth at each gene position divided by the total reads in the region, then averaged. **(C)** Distribution of coverage at all nucleotide positions within the CDS region (y-axis on a log10 scale). **(D)** Relationship between read length and the quality of mapped and unmapped reads basecalled by Dorado v5.1.0 model, presented as a 2D density plot. Read quality is indicated by Q scores.

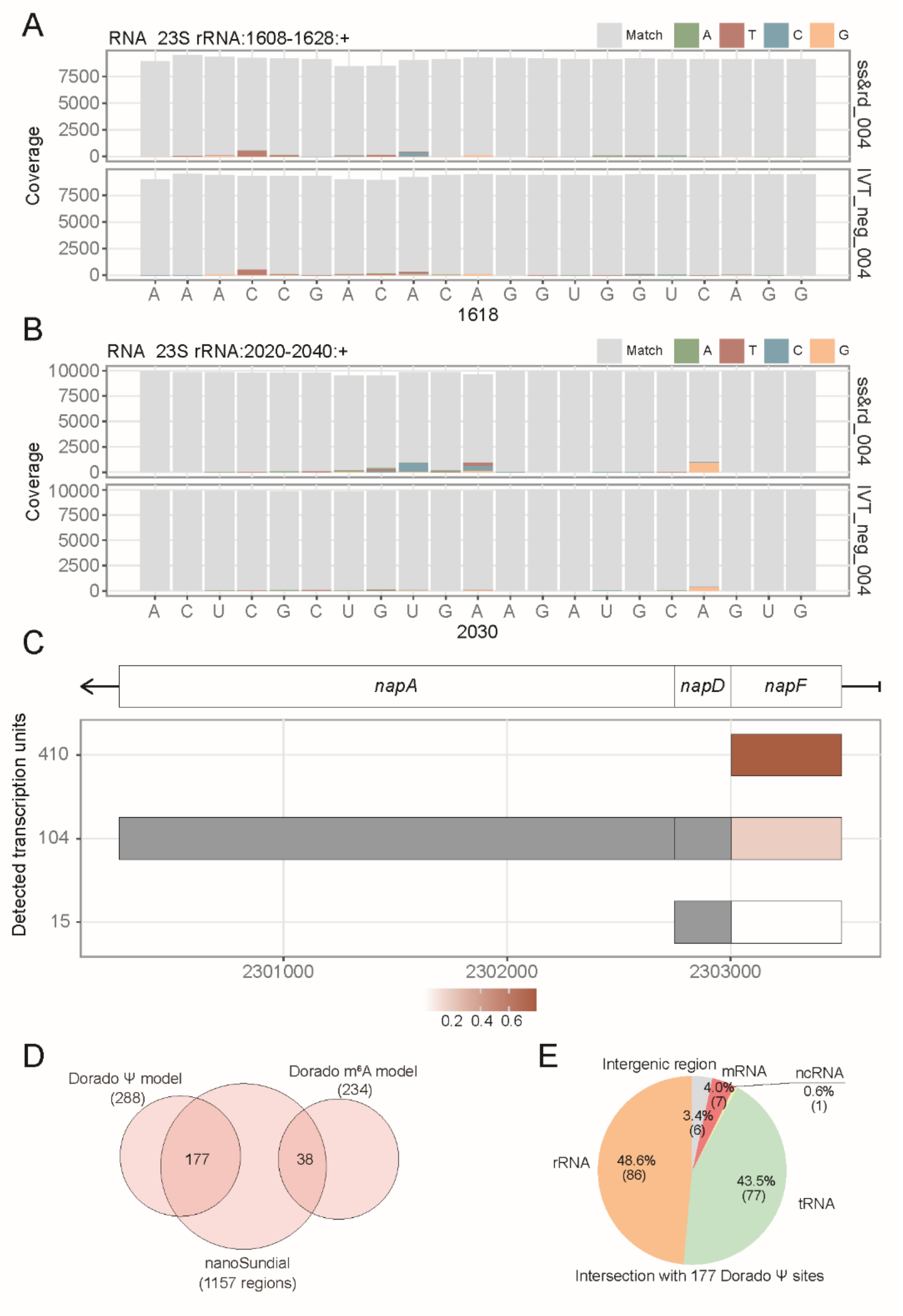

Figure S7. Exploratory analysis of nanoSundial performance and outcomes. Related to Discussion. **(A)** and **(B)** illustrate the matches and mismatches of A1618 and A2030, with the gray areas indicating the matching regions of each site. **(C)** The composition and proportion of the Transcription Units (TUs) for *napF*. The colors indicate the proportion of each TU relative to the total reads for one gene. **(D)** Overlap between positive regions identified by the Dorado model and nanoSundial. **(E)** RNA type distribution for the 177 sites jointly detected by the Dorado Ψ model and nanoSundial result.
